# The Anti-candidal and Absorbtion Performance of PVA/PVP-Based *Jania rubens* Hydrogel on *Candida tropicalis* and Some Physicochemical Properties of the Hydrogel

**DOI:** 10.1007/s12010-024-04997-1

**Published:** 2024-07-04

**Authors:** Meltem Boran, Elif Erdogan Eliuz, Deniz Ayas

**Affiliations:** Department of Seafood Processing Technology, Faculty of Fisheries, Mersin University, Mersin, Turkey

**Keywords:** *Jania rubens*, *Candida tropicalis*, Hydrogel, Anti-candidal activity

## Abstract

This study was aimed to create a bioactive hydrogel form with PVA/PVP (polyvinyl alcohol/poly(N-vinylpyrrolidone) polymer using acetone and ethanol extractions of *Jania* *rubens* red algae and investigate some pharmaceutical properties. The anti-candidal activity and some inhibition performance of *J. rubens*/PVA/PVP hydrogel were investigated on *Candida tropicalis* which is one of the important causes of bloodstream infections. The physicochemical properties of *J. rubens*/PVA/PVP hydrogel were revealed using FTIR and swelling-absorption tests. The volatile compounds of *J. rubens* extracts were examined by GCMS. By mixing the extracts in equal proportions, PVA/PVP-based hydrogel was prepared. According to the results, Cumulative Drug Release was stable at 25 °C for the first 5 h. The IZ (inhibition zone) and MIC (minimum inhibitory concentration) of *J. rubens*/PVA/PVP hydrogel were 9.01 mm and 80.20 mg/mL, respectively. It was found that logarithmic reduction and percent reduction were seen as 1.5 CFU/mL and 97.5%, respectively, on *C. tropicalis* exposed to *J. rubens*/PVA/PVP hydrogel in the first 5 min of the incubation. After exposure of *C. tropicalis* to *J. rubens*/PVA/PVP, the number of viable cells transferred from the gel to water was between 76.1 and 73.1% in high glucose medium, while it was between 92.2 and 80.8% for the PVA/PVP hydrogel under the same conditions. As a result, PVA/PVP hydrogel was made bioactive with *J. rubens* extracts for the first time in this study, and its potential for use as a functional anticandidal hydrogel on *C.* *tropicalis* has been demonstrated.

## Introduction

*Jania rubens* is a member of the Corallinaceae family of red algae (Rhodophyta). It spreads in the Mediterranean-Black Sea, North-East Atlantic, Indian Ocean, and China Sea [1]. *J. rubens* are distinguished from other algae by the absence of flagella in their sexual reproduction. The thalli are multicellular, and the basis of the thallus structure is filamentous. The wall of thallus cells is made of cellulose and various pectic compounds. The inner layer is cellulose, and the outer layer is mucilage pectin. Many studies have shown that *J. rubens* is used in treating some diseases and is responsible for many biological activities due to its active ingredients of fatty acids, alkanes, sterols, vitamins, trace elements, halides, mannitol, and some proteins. In ancient times, it was used to treat intestinal ulceration and radioisotope poisoning, and it stated that it benefited from its hypoglycemic, fibrinolytic, and lipolytic activities [2–4]. Another biological activity of the algae is its antimicrobial potential due to secondary metabolites in its content. In previous studies, Ismail-Ben Ali et al. reported that dichloromethane and dichloromethane/methanol extracts of *J. rubens* were active on *Staphylococcus aureus*, and *Micrococcus* sp. [5]. It was found that *J. rubens* had the highest antibacterial activity against *S. aureus* [6]. It has been reported that methanol, dichloromethane, hexane, chloroform, and essential oil extracts of *J. rubens* are highly effective against *Streptococcus faecalis*, *Bacillus subtilis*, *S. aureus*, *S. epidermidis*, *Escherichia coli*, *Salmonella typhimurium*, and *Candida albicans* microorganisms [1].

*C. tropicalis* is a member of the kingdom Fungi, of the division Ascomycota, and genus *Candida*. The frequency of spread varies depending on the geography where the invasive disease occurs and causes 3–66% of candidemia [7]. Studies have reported that after infecting the patient, *C. tropicalis* causes rapid spread and high mortality rate, especially in patients with weakened immune systems [8, 9]. In recent years, *Candida tropicalis* has been the leading non-albicans *Candida* species causing nosocomial fungal bloodstream infections [10]. This has led to the idea that *C. tropicalis* is responsible for 50% of *Candida* infections. Geographical structure and epidemiological changes have shown that *Candida glabrata* replaced *C. tropicalis* in some cases [11]. In a recent study, an increase in the incidence of bloodstream infections caused by *Candida parapsilosis* and *Candida tropicalis* was observed during the Coronavirus disease 2019 epidemic [12]. *Candida tropicalis* is one of the most known *Candida* species in terms of its virulence and drug resistance. One of the most important reasons for this is the ability of yeast to adhere to surfaces and form biofilms [13]. It is often resistant to the immune system cells of the host it infects and likely to serve as a reservoir for persistent sources of infection [14]. Compared to other *Candida* species, the strong biofilm-forming ability of *C. tropicalis* provides resistance to the varying pH and osmolarity range in the environment [15]. Therefore, they are highly resistant to antifungal treatments and cause dangerous infections with species such as *C. albicans* and *C. glabrata* [16, 17]. Treatment with *C. tropicalis* has been reported to produce strains increasingly resistant to fluconazole and other antifungal agents. This highlights the need to investigate new alternative therapeutics with antifungal effects [18, 19].

Hydrogels are semi-fluid, soft, or slightly rigid structures of cross-linked hydrophilic polymeric networks. Various hydrogels can be formed depending on the crosslinking points and mechanical strength of three-dimensional mesh. They can be divided into several classes based on their source, method of preparation, electrical charge, configuration, crosslinking, and function [20]. Some of these include physical hydrogels containing hydrogen bonds, van der Waals and hydrophobic interactions, and chemical hydrogels with covalent bonds [21]. Hydrogels developed by many chemical and physical methods have found application in tissue engineering, pharmaceutical, and biomedical fields [22]. Especially, hydrogels have significant advantages in wound care and other pharmaceutical treatments. These include features such as keeping humidity constant and reducing pain due to the cooling effect in the microenvironment [23]. Polyvinyl alcohol (PVA) and poly(N-vinylpyrrolidone) (PVP), which are widely used in hydrogel synthesis, are a group of polymers that are biocompatible and have extremely low cytotoxicity [24, 25]. PVA/PVP-based hydrogels developed for areas such as wound treatments and drug delivery devices can be processed using different methods such as freeze–thaw and γ-irradiation techniques [26, 27].

Plant-based hydrogels can be used as hydrogel sources because they are natural, harmless, and renewable [27]. Among them, cellulose-based hydrogels [28] are the most widely known. Hydrogels based on starch [29], pectin [30], or soy protein-polysaccharide [31] have biomedical and food applications. Functional hydrogels with different properties are developed using algal resources. The primary material of algae-based hydrogels was mostly polysaccharides (alginate, carrageenan, starch, agarose, porphyrin, and cellulose) [32]. Apart from these, we rarely come across hydrogels containing extracts prepared to reveal bioactive components of algae such as phenolic and volatile components. For example, *Chlorella vulgaris* green algae were extracted in dry form in TBS buffer (Tris–HCl-saline, pH 7.5), and this extract, which contains various bioactive compounds, was hydrogelized with the carbopol agent [33]. In another study, the extraction of *Cladophora glomerata* using acetone, methanol, and ethanol solvents was investigated, and various glomerata hydrogels were developed using GCMS analysis and subsequent extracts. The study emphasized that this hydrogel could potentially be developed as a skin care product [34]. In another study, polyamidoamine-based hydrogels containing photoactive chlorophyll a obtained from Spirulina seaweed extract were developed for potential applications in photodynamic therapy [35]. Red algae, which contain various photoprotective and antiphotoaging compounds, are organic sources that will enrich hydrogels with bioactive molecules [36]. Two anthocyanins (malonylchisonine and 4′-demalonylsalvianin), found in *J.* *rubens* extract and belonging to the flavonoid class, are important components known to provide anti-aging and protection against UV [37]. In another study, the volatile components n-docosane, n-eicosane, and n-tetratriacontane (5.58%) were detected [1]. However, there are no studies in which these compounds are integrated into hydrogel structures.

In this study, anti-candidal activity and the time-dependent death dynamic against *Candida tropicalis* of *J. rubens*/PVA/PVP hydrogel were reported for the first time. The characteristic features of this hydrogel and the volatile content of the mixed extract were revealed using GCMS and FTIR, respectively.

## Experimental Sections

### Preparation of *J. rubens* Ethanol and Acetone Extractions

*J. rubens* was collected from Mersin Akkum beach (38.193003, 26.770186) in Turkey (2020). *J. rubens* samples were brought to Mersin University, Faculty of Fisheries laboratory with the help of a cold chain. Identification of *J. rubens* was made by Prof. Dr. Deniz Ayas. The extraction method was studied by modifying traditional methods to reveal bioactive components of *J. rubens* [38, 39]. The sand on the *J. rubens* surface was cleaned by several washes, left to dry, and ground in the robot. In preparation for the extraction, approximately 4 g of *J. rubens* sample was soaked in 40 mL of ethanol and acetone solvents separately for 1 day and then stirred on a magnetic stirrer for 2 h. Then, the sample (0.1 g/mL) was filtered, and the filtrate was passed through a sterile 0.45 µm filter and stored at 4 °C to be used in the experiments.

### Analysis of Total Phenol in *J. rubens* Extracts

The total phenol content of *J. rubens* extractions was investigated using the Folin–Ciocalteu method applied by Erdoğan Eliuz [40]. Each extraction was performed in 3 parallels. First, 0.5 mL of Folin–Ciocalteu reagent and 0.5 mL of sample solutions were placed in the tubes and mixed. It was kept in the dark for 5 min, and then, 2 mL of Na_2_CO_3_ (200 g L^−1^) solution and 3 mL of distilled water were added, remixed, and kept in the dark for 30 min. Absorbance measurements were made at 700 nm using a UV spectrophotometer. The gallic acid standard was used to calculate the concentrations of the extracts. The data obtained are given as milligrams of gallic acid (mg GAE/100 g dw) [40].

### Analysis of Volatile Compounds in *J. rubens* Extracts by GC–MS

GC–MS (Gas Chromatography-Mass Spectrometry) analysis of *J. rubens* extracts were performed using a 7890A GC-5975C MSD (Agilent) instrument and HP-5MS column (30 m × 250 µm × 0.25 µm). In the analysis, helium with a flow rate of 1 mL/min was used as the carrier gas, and the furnace temperature was started at 50 °C, kept at this temperature for 3 min, and increased to 300 °C with 10 °C/min increments per minute and kept at this temperature for 6 min. The injection volume for each sample is 1 μL, and the ionization voltage is 70 eV. Separated components were evaluated by comparing them with NIST 2008 (National Institute of Standards and Technology) and National Standards Institute data [41].

### Preparation of *J. rubens*-Based PVA-PVP Hydrogel

Freeze–thaw method was used to prepare the *J. rubens-based* PVA-PVP hydrogel. PVA (20 wt%) and PVP (12 wt%) were dissolved in doubly distilled water and heated in a water bath at 90 °C for 5 and 15 min, respectively. After the temperature of each solution reached approximately 90 °C, PVA/PVP in different compositions were mixed. Then, 5%, 10%, or 20% of *J. rubens* extracts (*J. rubens* ethanol and acetone extracts in v/v ratio) were added, separately. The polymer solutions were stirred under magnetic stirring for 2 h at room temperature. The mixture was poured into Petri plates and kept directly frozen for 16 h at − 18 °C. The frozen hydrogels were then thawed at room temperature for 8 h. This freezing/thawing process to crosslink the polymer was repeated three times [22]. Among the prepared hydrogels, analyses were continued with hydrogels that are effective against *C. tropicalis* in liquid form (by testing disc diffusion test method). In this study, PVA/PVP hydrogel formed with a 20% extract (100 mg/mL) mixture was studied.

### Swelling Measurements of *J. rubens*/PVA/PVP Hydrogel

*J. rubens* hydrogel, completely dried in the lyophilizer, was weighed on a precision balance and then completely immersed in 200 mL of distilled water. It was left at room temperature for 3 h and hung for 10 min to remove excess water. Equilibrium swelling (ES) was calculated according to Eq. 1.

1 $$\text{ES}(\text{\%})=\frac{W2-W1}{W1}*100$$

*W*1 and *W*2 are the weights of dry and swollen gel, respectively.

### FTIR Analysis of *J. rubens*/PVA/PVP Hydrogel

The FT-IR (Fourier Transform Infrared Spectrophotometer) spectra of the *J. rubens* hydrogel were analyzed using FT-IR (Brand: Jasco FT/IR-6700) in ATR mode, with a spectral measurement range of 4000–500 cm^−1^. In the hydrogel, bioactive compounds based on aromatic, phenyl, and acyl groups were determined according to their FT-IR spectra [42].

### Determination of IZ and MIC of *J. rubens*/PVA-PVP Hydrogel on *C. tropicalis*

Before testing, the yeast was inoculated on SDA (Sabouraud dextrose agar) solid medium and incubated at 37 °C for 18–24 h. At the end of 1-day incubation, colonies were taken directly from the single fallen colonies on the agar plate with the help of a loop, and the McFarland (~ 10 CFU/mL) was adjusted with saline. Fluconazole was used for yeasts as a positive control antibiotic.

According to the disc diffusion method, a certain amount of microorganism solution adjusted according to McFarland 0.5 was spread on the Petri dish with MHA agar, and 6 mm diameter discs were added in the middle of the Petri plate. Each disc was filled with 20 µL of liquid form of *J. rubens*/PVA/PVP and incubated at 37 °C for 24 h. When evaluating the results, the diameters of the (IZ) were measured in millimeters using the Images program. To calculate the MIC, the double dilution concentrations of the hydrogel were studied as in the mentioned experiment [40]. A graph of the extract concentration (%) and  inhibition zone was plotted with the results. The % inhibition was calculated using the following formula in Eq. 2. Anti-candidal activity of *J. rubens*/PVA/PVP was compared with PVA/PVP hydrogel and *J. rubens* extracts as a negative control, and all tests were repeated thrice.

2 $$\text{Inhibition }(\text{\%}) =1-[\frac{\text{OD test IZ}}{\text{OD corresponding control IZ}}]\times 100$$

#### In Vitro Cumulative Drug Release

0.1 g of *J. rubens*-loaded hydrogel was weighed and added to 100 mL of PBS (7.4) as a release medium. Triplicate samples were shaken in a shaking incubator at 80 rpm for 8 h. Samples taken at certain time intervals were filtered through a 0.45 µm membrane filter and replaced with fresh medium. The results were measured by the spectrophotometric (280 nm) method as a function of time. The averages of the experiments were taken, and a graph was created according to the formula in Eq. 3 [43].

3 $$\text{Cumulative release }(\text{CR})=\left(\frac{Mt}{M\infty }\right)*100$$

*Mt* is the amount of *J. rubens* mix-extract released from the hydrogel at time *t* and *M*∞ is the estimated amount of *J. rubens* mix-extract loaded into the hydrogel.

#### Time-Related Dynamics of Mortality of *C. tropicalis* on Exposure to *J. rubens*/PVA/PVP Hydrogel

Five microliters of *C. tropicalis* inoculum (McFarland 0.5) was transferred into the tubes previously added with *J. rubens*/PVA/PVP hydrogel (50 µL) and vortexed for 5 min. The same procedure was also prepared for negative control with PVA/PVP hydrogel. All samples were incubated at 37 °C at certain time intervals (5, 15, 25 min) in a shaking incubator. Afterward, the samples were taken out of the incubator and taken into sterile glass tubes containing 1 mL of saline and shaken for 5 min in a shaking incubator to allow the yeasts to pass into the water. After 10^−2^ serial dilutions of the samples, 5 µL of sample from the tube was inoculated into MHA plates and incubated at 37 °C for 24 h. After 1 day, colonies were counted visually, and logarithmic reduction and percent inhibition (Eqs. 4 and 5). Petri dishes in which more than 300 colonies were counted were not taken into account in the experiments, and experiments were performed three times for each time interval [44].

4 $$\text{Percent reduction }\%=\left[\frac{ODx}{ODc}\right]*100=\frac{\left(A-B\right)\times 100}{A}$$

5 $$\text{Logarithmic decrement }=\text{Log}10\frac{A}{B}=\text{Log}10(\text{A})-\text{Log}10(\text{B})$$

#### Testing of *J. rubens*/PVA/PVP as an Adsorbent of *C. tropicalis*

The TTC-DRA method is a method used to determine the viability of microbial cells after treatment with antimicrobial agents. The dehydrogenase enzyme found in living cells produces insoluble red 2,3,5-triphenyl formazan (TF) and is reduced by the hydrogen acceptor TTC dye molecule in microbial culture. Therefore, to determine TTC-DRA in cells, the absorbance value of the TF molecule is measured at 485 nm. In this study, the absorption of *C. tropicalis* cells by *J. rubens*/PVA/PVP hydrogel was evaluated. Hydrogel cut to approximately 1 cm^2^ in size was combined with *C. tropicalis* (McFarland 0.5) culture (1 mL) under aseptic conditions and vortexed. After waiting for 30 min, the gel was removed from the yeast solution and transferred to a 1 mL water medium containing TTC dye (0.01 mg/mL) and glucose (0.01 or 3 g/L to mimic a clean and dirty organic environment condition). All samples were kept in the incubator for 8 h to stain live cells. Then, all tubes were vortexed for 5 min to move the attached cells into the water and then kept at 37 °C for 15 min. This process was repeated 3 times. Live yeast cells transferred to water were measured spectrophotometrically. PVA/PVP hydrogel (non-glucose) was used for control. The DRA (dehydrogenase activity) is formulated as follows (Eq. 6):

6 $$\text{DRA }(\text{\%}) = \frac{Dx}{Dc}*100$$

where ODx and ODc represent the absorbance of the treated and control samples, respectively.

### Statistical Analysis

MIC, IZ, and Log red. analyses and significance were tested using One-way ANOVA with post-hoc Tukey HSD Test (*p*-value < 0.05).

## Results and Discussions

### Total Phenol Content and GCMS Analysis of *J. rubens* Extracts

The total phenolic contents of the extracts were determined as 0.002 mgGAE/100 for *J. rubens* ethanol and 0.004 mgGAE/100 for acetone on a dry weight basis, respectively. In general, it is stated that there are phenolic compounds in the range of 1.5–4.1 mg GAE/g in crude methanolic extracts of red seaweed [3]. Phenolic compounds are commonly found in plants, including seaweeds, and have been reported to exhibit a wide variety of biological activities, including antimicrobial properties. Reports revealed that phenolic compounds are the most effective compounds in red algae. These compounds are known to be critical metabolites responsible for antimicrobial activity [45].

### GC-MS Analysis of *J. rubens* Extracts

Extraction of secondary metabolites found in plants is difficult due to their insoluble nature. In the literature, solvents such as methanol, ethanol, acetone, n-hexane, isooctane, and ethyl acetate are used effectively in secondary metabolite extractions. It has been reported that ethanol and acetone are effective in dissolving tannins, polyphenols, flavonols, terpenoids, and alkaloids [46, 47]. In our study, we used ethanol and acetone for the detection of volatile compounds of *J. rubens*. According to the GC-MS analysis, while the main compounds of *J. rubens *EE were phenol, 2,4-bis(1,1-dimethylethyl) (25.64%), 4.alpha.,7,8a.beta.-Trimethyl-3,4, 4a in ethanol extract. as beta.,5,6,8a-hexahydronaphthale n-1(2H)-one (3.99%), hexadecanoic acid, methyl ester (14.59%), ethyl palmitate (5.14%), oleic acid, methyl ester (6.43%), the major compounds of *J. rubens *AE were diacetone alcohol (85.57%), ethyl palmitate (3.11%), octadecanoic acid, and ethyl ester (6.04%) (Table 1).

**Table 1:** GCMS analysis of *J. rubens* AE and EE extracts

Rt	Compound	*J. rubens* EE		*J. rubens* AE	
		%	Q	%	Q
4.197	Diacetone alcohol	**-**	**-**	85.57	83
14.483	Phenol, 2,4-bis(1,1-dimethylethyl)				
	25.64	96	**-**	**-**	
24.490	4.alpha.,7,8a.beta.-trimethyl-3,4,4a.beta.,5,6,8a-hexahydronaphthale n-1(2H)-one	3.99	78	**-**	**-**
26.804	Hexadecanoic acid, methyl ester				
	14.59	99	**-**	**-**	
27.825	Ethyl palmitate				
	5.14	99	3.11	98	
29.226	Oleic acid, methyl ester				
	6.43	93	**-**	**-**	
30.276	Octadecanoic acid, ethyl ester				
	**-**	**-**	6.04	99	

*RT* retention time

In the literature, Karabay-Yavasoglu et al. identified 40 compounds as a result of GC–MS analysis of volatile components of *J. rubens* in their study [1]. The main volatile components of *J. rubens* were n-docosane (6.35%), n-eicosane (5.77%), and n-tetratriacontane (5.58%). In addition, this extract did not affect *Candida albicans* growth, and the IZ was found as zero. In another study, The GC–MS analysis of the crude extract of *J. rubens* revealed that the main chemical constituent was 1-( +)-ascorbic acid 2,6-dihexadecanoate (35.48%) followed by icosapent (6.91%), trans-13 octadecenoic acid (5.04%), 3,7,11,15-tetramethyl-2-hexadecen-1-ol (4.66%), heptadecane (4.01%), and 1,2-benzenedicarboxylic acid (3.10%) [3]. Phenol, 2,4-bis(1,1-dimethylethyl) was found in fermented seaweeds and Kappaphycus spp. red algae enhanced with endophytic mushroom fermentation [48, 49]. In this study, phenol, 2,4-bis(1,1-dimethylethyl) substance in *J. rubens* EE (25.64%) could limit the antimicrobial activity of microorganisms other than *C. tropicalis*. Because, it has been reported that the related compound acidified the environment and caused the reduction of reactive oxygen species (ROS), which causes the inhibition of pathogens [50]. Methyl palmitate, which is found in significant amounts in *J. rubens* EE, has been previously detected in *Ulva australis* [51]. Contrary to other studies, compounds such as 2,4-bis(1,1-dimethylethyl), hexadecanoic acid, methyl ester (14.59%), and ethyl palmitate detected in this study may have affected *Candida tropicalis*.

### Characteristic Properties of *J. rubens*/PVA/PVP Hydrogel

**Fig. 1:**
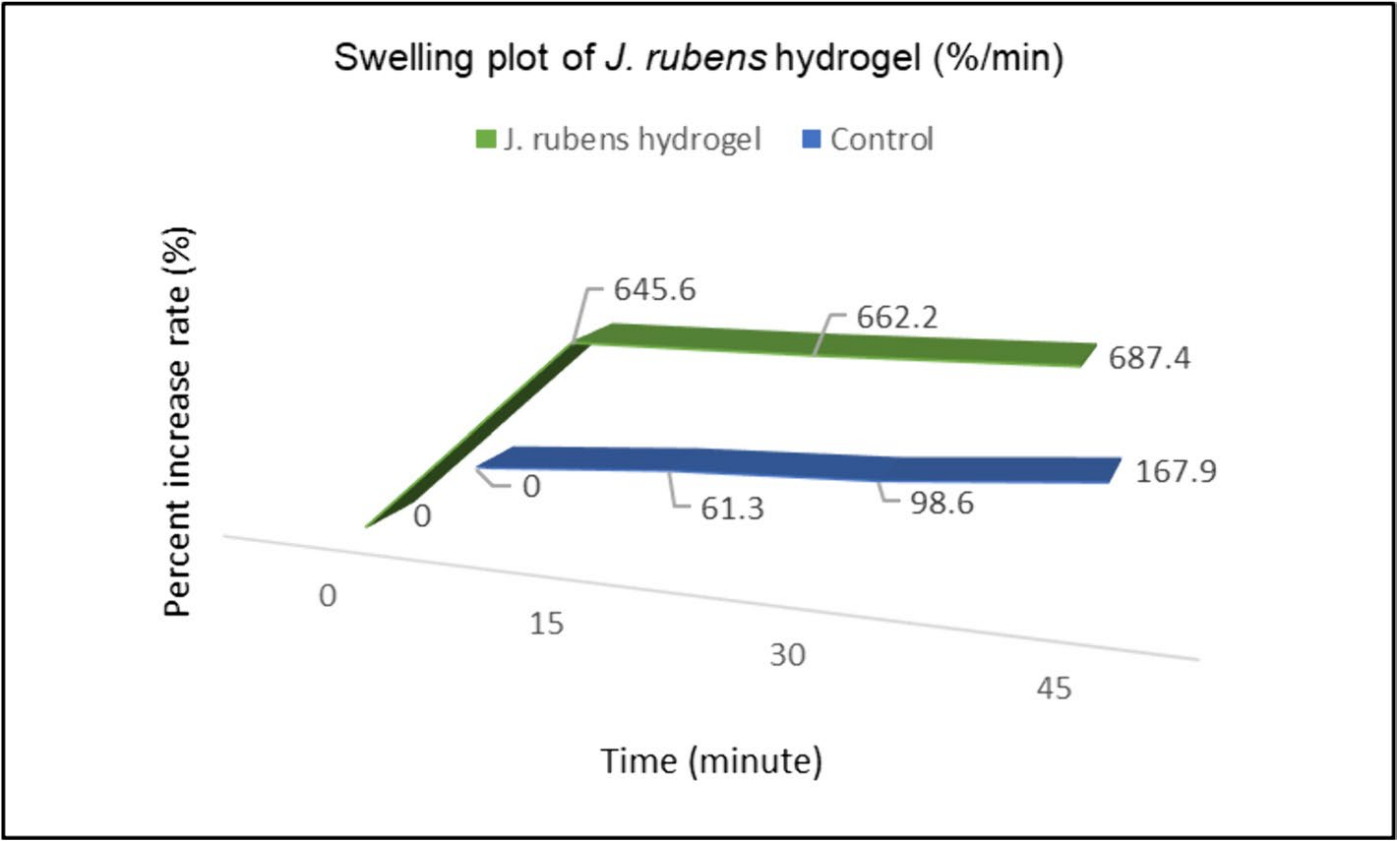
Swelling rate (%) on *J. rubens*/PVA/PVP hydrogel and control (PVA/PVP hydrogel)

The time-dependent swelling property of *J. rubens*/PVA/PVP hydrogel is shown in the graph (Fig. 1). When this graph was examined, it was observed that the swelling behavior of *J. rubens*/PVA/PVP reached its maximum level in the first 15 min with a 645.6% increase rate. Afterward, the swelling rate slowed down and remained stable for 45 min. The initial weight of the control hydrogel (PVA/PVP) was 0.0428 g, while the initial weight of *J. rubens*/PVA/PVP hydrogel was 0.0949 g. This difference comes from the algae extract added from the algae in the *J. rubens*/PVA/PVP gel.

**Fig. 2:**
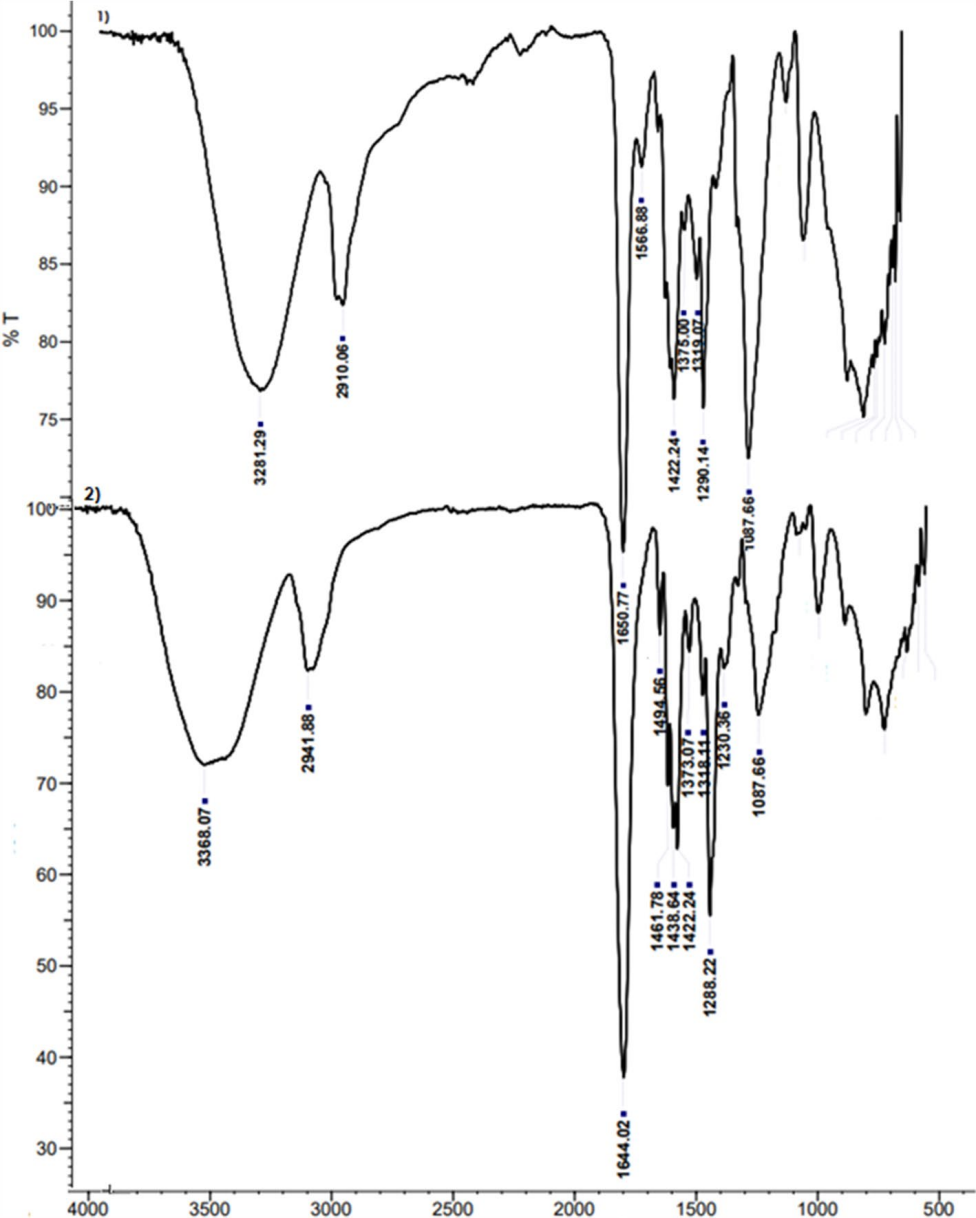
IR spectra of PVA/PVP (**1**) and *J. rubens*/PVA/PVP hydrogel (**2**). T (%): transmittance

The IR spectra of the PVA/PVP (Fig. 2-1) and *J. rubens*/PVA/PVP hydrogel (Fig. 2-2) were shown in the wavenumber range of 4000 to 400 cm^−1^. In the FT-IR of pure hydrogel alone, 3281.29 cm^−1^ (O–H groups; stretch modes in water and hydroxyl), 2910.06 cm^−1^ (alkyl groups), 1650.77 cm^−^^1^ (C = O), 1566.88 cm^−1^, 1422.24 cm^−1^ (C-H vibration), 1375.00 cm^−1^ and 1319.07 cm^−1^ (CH_3_ vibration), 1290.14 cm^−1^, and 1087.66 cm^−1^ (C-O stretching) peaks were detected. Fusing PVP with PVA is 3281.27 cm^−1^, where the O–H stretch of PVA is high. C = O stretching of PVP was seen from 1660 cm^−1^ to 1655–1651 cm^−1^. These results demonstrate the intermolecular hydrogen bonding between the hydroxyl groups of PVA and the carbonyl groups of PVP. These results are consistent with previous reports [52]. All major peaks related to hydroxyl and acetate groups were observed in the FTIR spectra of PVA. The large bands observed between 3550 and 3200 cm^−1^ are due to O–H extending from intermolecular and intermolecular hydrogen bonds. The vibration band followed between 2840 and 3000 cm^−1^ is due to alkyl groups. Mixture gel of PVA and PVP shows that hydrogen-bonded single bond OH groups (at about 3200–3500 cm^−1^) and C = O peak shifts (about 1650–1680 cm^−1^) and dominant intermolecular interactions in PVA occur [52]. In the FT-IR of *J. rubens* hydrogel, bands of 3368.07 cm^−1^ (phenol), 2941.88 cm^−1^ (lipid acyl chain), 1644.02 cm^−1^ (aromatic C–C stretch), 1494, 56 cm^−1^ (phenyl groups), 1461.78 cm^−1^ (C-H vibration), 1438.64 (C-H vibration), 1422.24 (C-H vibration), 1373.07 cm^−1^ and 1318.11 cm^−1^ (CH_3_ vibration), 1288.22 cm^−1^ (C-O stretching vibrations), 1230.36 (C-N vibrations), and 1087.66 cm^−1^ (C-O; C–C and C-H) were found. Bands at and around 1400 cm^−1^ of the hydrogel indicate that the gel contains many residual organic groups.

### Cumulative Drug Release

**Fig. 3:**
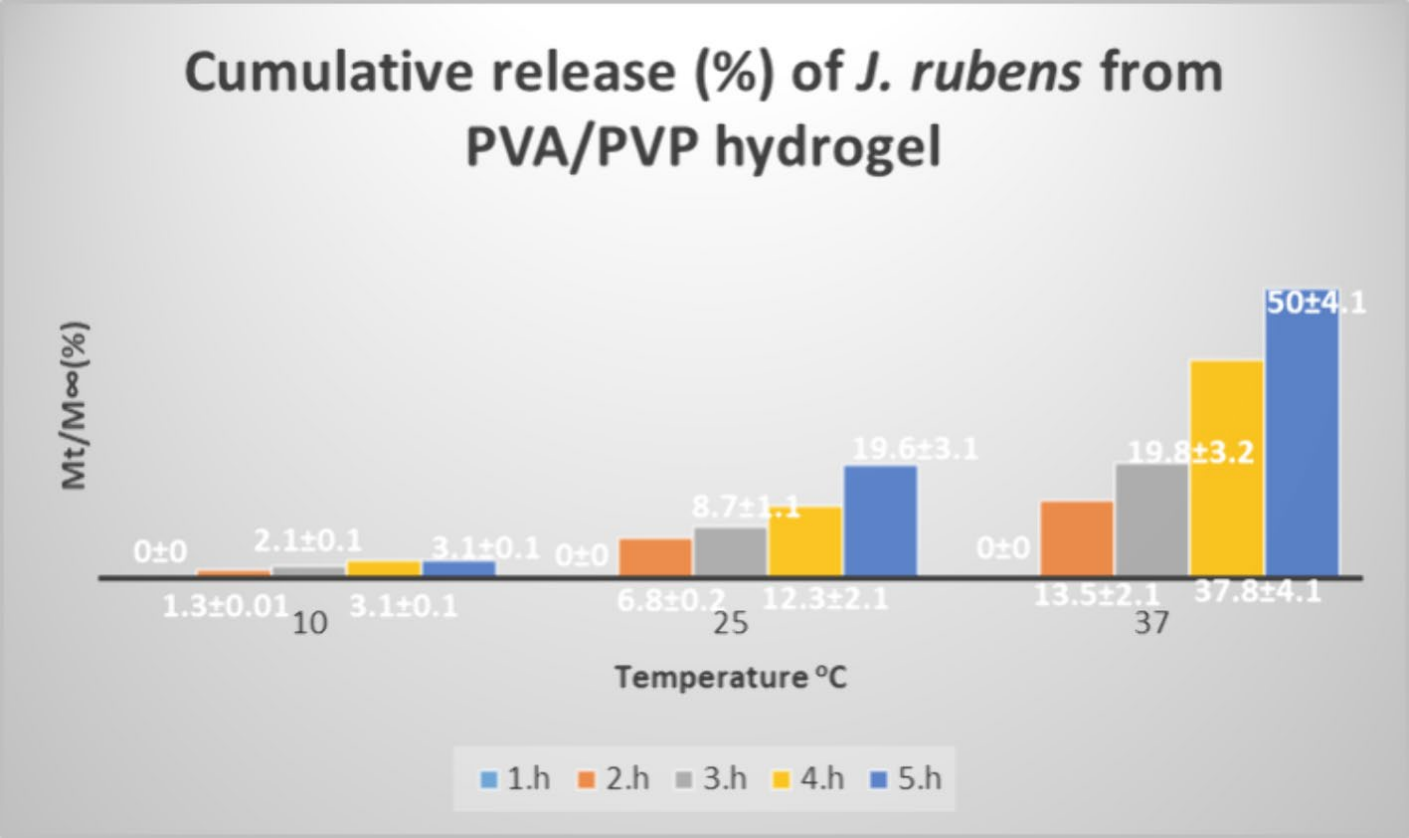
Cumulative release of *J. rubens* from PVA/PVP hydrogel at 10 °C, 25 °C, and 37 °C in buffer solutions of pH 7.4

As shown in Fig. 3, the release of *J. rubens* from PVA/PVP hydrogels increased with increasing temperature. This shows that the spreading of *J. rubens/*PVA/PVP will accelerate with temperature. At 37 °C, the maximum release was observed from the 1st hour to the 5th hour, and the order is as follows: 0%, 13.5%, 19.8%, 37.8%, and 50% respectively (*p*-value ≤ 0.05). There is a significant oscillation after the 3rd hour at 25 °C. These are 8.7%, 12.3%, and 19.6% respectively. At 10 °C, no significant oscillation was observed in the first 5 h (*p*-value ≤ 0.05).

### Anti-candidal Activities of *J. rubens*/PVA/PVP Hydrogel

**Fig. 4:**
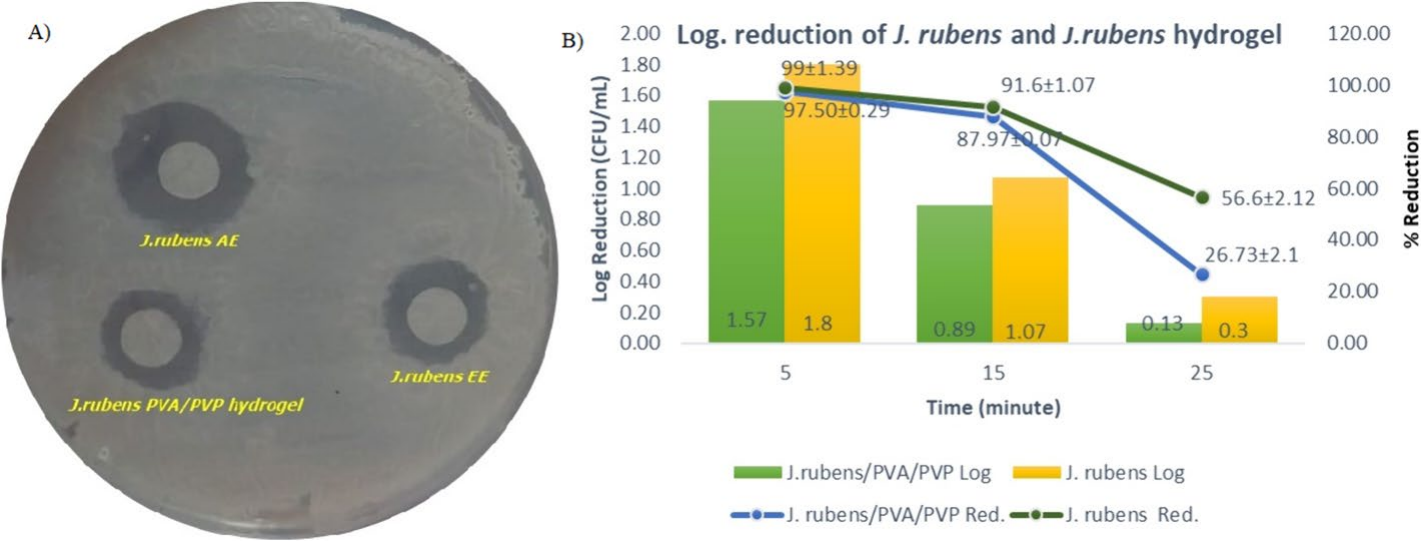
Inhibition zones of *J. rubens* EE, *J. rubens* AA, and *J. rubens*/PVA/PVP (**A**); time-dependent death dynamics of *C. tropicalis* exposed to *J. rubens*/PVA/PVP hydrogel (**B**)

Antimicrobial activities of *J. rubens****/***PVA/PVP hydrogel against *C. tropicalis* were tested (*p*-value < 0.05). The results obtained are given in Table 2. The IZ and MIC of *J. rubens****/***PVA/PVP hydrogel were 9.01 mm and 80.20 mg/mL, respectively. The ethanol and acetone extractions of *J. rubens* showed inhibition zones of 10.14 mm and 12.37 mm against *C. tropicalis* (*p*-value < 0.05) (Fig. 4A). The MIC values of *J. rubens* ethanol and acetone extracts were determined as 91.5 mg/mL and 99.25 mg/mL, respectively (*p*-value ≤ 0.05).

**Table 2:** IZ diameters (mm) and MIC values (mg/mL) of *J. rubens*/PVA/PVP and *J. rubens* EE and AE against *C. tropicalis*

	*J. rubens*/PVA/PVP	PVA/PVP	*J. rubens* EE	*J. rubens* AE	Fluconazole (120 µg/mL)
IZ (mm)	9.01^a^ ± 0.1	0	10.14^a^ ± 0.1	12.37^a^ ± 0.1	30.0 ± 0.02 µg/mL
MIC (mg/mL)	80.20^b^ ± 2.1	0	91.5^b^ ± 3.7	99.25^b^ ± 3.7	25 ± 0.12 µg/mL

Statistical differences are indicated by a different letter in each line (*p*-value < 0.05)

Antifungal activities of *J. rubens* have been reported in several previous publications [1, 5, 53]. However, there are rare publications about the antifungal activities of *J. rubens* on *Candida tropicalis*. The extracts of methanol, diethyl ether, ethyl acetate, petroleum ether, ethanol, and acetone of *J. rubens* highly affected *C. tropicalis* between 10 and 30 mm [54, 55]. Polyhydroxysteroids such as cholesta-8-en-3β,5α,6α,25-tetrol and cholesta-8(14)-en-3β,5α,6α-25-tetrol extracted from another algae species (*Lamellodysidea herbacea*) were inhibited to *C. tropicalis* with 13 and 11 mmat 10 mg/disc [56]. These results were similar to our data (≥ 10 mm).

### Dynamics of Time-Related Death of *C. tropicalis* When Exposed to *J. rubens*/PVA/PVP

In this study, the microbicidal effect of *J. rubens*/PVA/PVP hydrogel was determined dynamically over time (Fig. 4B). In the first 5 min, of incubation of *C. tropicalis* fungus exposed to the hydrogel, logarithmic reduction and reduction were 1.5 ± 0.12 CFU/mL and 97.5% ± 0.7, respectively. It was calculated as 0.89 ± 0.15 CFU/mL and 87.97 ± 3.6% at the 15th minutes and 0.13 ± 0.12 and 26.73% ± 23.11% at the 25th minutes. In the study, it can be concluded that *J. rubens*/PVA/PVP hydrogel affected *C. tropicalis*, significantly, in the first 5 min (*p* ≤ 0.5).

#### Testing of *J. rubens*/PVA/PVP as an Adsorbent of *C. tropicalis*

The following study was conducted to understand the viability of *C. tropicalis* cells exposed to *J. rubens* hydrogel surface compared to the control. Penetration and inhibition of *Candida* on the *J. rubens* hydrogel surface were expected when *Candida* cells were compared to the bioactive substances in the hydrogel. Cells that survived and migrated into water were measured spectrophotometrically. The density of living cells adhering to *J. rubens*/PVA/PVP hydrogel and then passing into water was found to be higher than PVA/PVP hydrogel alone. The rate of live cells of *C. tropicalis* absorbed to *J. rubens*/PVA/PVP was 75.2%, 66.2%, 60.2%, 60.1%, and 60% in low glucose environment, respectively, while they were 76.1%, 75.1%, 75.1%, 74%, and 73.1% in high glucose environment within 5, 20, 40, 80, and 120 min, respectively. While PVA/PVP hydrogel absorbed *C. tropicalis* cells at the level of 91.2%, 81.5%, 80.2%, 80%, and 80% in low glucose environment, they were 92.2%, 87.5%, 82.2%, 80.9%, and 80.8% in high glucose environment within 5, 20, 40, 80, and 120 min, respectively. In this case, we can say that the fungal absorption capacity of phenolic gel is higher (Table 3).

**Table 3:** TTC-DRA (%) of *C. tropicalis* after *J. rubens*/PVA/PVP and PVA/PVP hydrogels treatment in low-glucose (0.01 g/mL) and high-glucose (3 g/L) environmental conditions

Time (min)	DRA (%)			
	*J. rubens*/PVA/PVP		PVA/PVP	
	Low-glucose	High-glucose	Low-glucose	High-glucose
5	75.2^a^ ± 0.1	76.1^a^ ± 0.1	91.2^a^ ± 0.1	92.2^a^ ± 0.1
20	66.2^a^ ± 0.1	75.1^a^ ± 0.1	81.5^a^ ± 0.1	87.5^a^ ± 0.1
40	60.2^a^ ± 0.1	75.1^a^ ± 0.1	80.2^a^ ± 0.1	82.2^a^ ± 0.1
80	60.1^a^ ± 0.1	74^a^ ± 0.1	80^a^ ± 0.1	80.9^a^ ± 0.1
120	60^a^ ± 0.1	73.1^a^ ± 0.1	80^a^ ± 0.1	80.8^a^ ± 0.1

Statistical differences are indicated by a different letter in each column (*p*-value < 0.05)

**Fig. 5:**
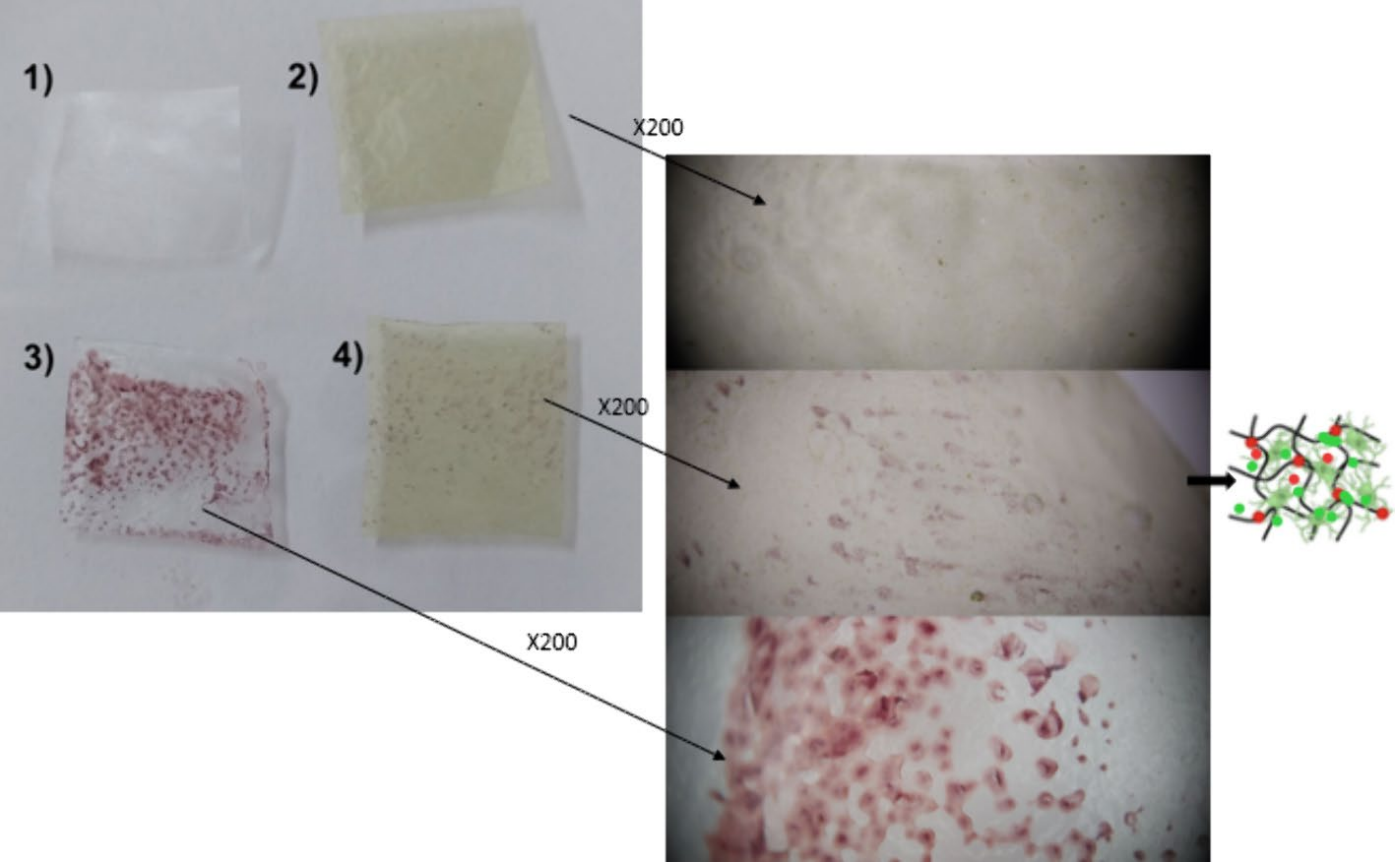
Absorbance of *C. tropicalis* by the gels. PVA/PVP free gel (1-control), *J. rubens*/PVA/PVP free gel (2), cell loaded PVA/PVP, and *J. rubens*/PVA/PVP (3 and 4, respectively)

When hydrogels are cross-linked with their parent material, they form an insoluble three-dimensional structure that increases water absorption capacity [57]. The ability to rapidly absorb water supports biocompatibility, which is an advantage in pharmaceutical, cell delivery systems, or antimicrobial research. Hydrogel formulations containing methylcellulose [58], Ginja cherry extract [59], clove bud (*Syzygium aromaticum*), olive leaf extract [60], and thyme (*Origanum vulgare*) [61] essential oils have been synthesized due to their ability to absorb water and antimicrobial activity. Among these, methylcellulose has been recommended for use in oral mucosa and/or vaginal applications due to its ability to adhere to mucosal surfaces [62, 63]. In this study, it appears that *J. rubens*-based hydrogel rapidly absorbs the microorganism together with water in the water environment where *Candida* is present (Fig. 5). The presence of fewer *Candida* cells in the *J. rubens*/PVA/PVP hydrogel compared to the control surface (PVA/PVP) may be associated with the inhibition of *C. tropicalis* by the phenolic extract. In vivo studies are needed to make hydrogel forms of bioactive substances from *J. rubens* ethanol or acetone extracts applicable. In particular, acetone is mentioned to be limited in use due to toxicity. However, a few studies have shown that acetone can also be used in in vivo applications at certain concentrations (< 20 mmol/kg) [64, 65].

## Conclusion

*Candida tropicalis* has been inhibited by many algae extracts in the literature including *J. rubens*. This study tested the effectiveness of *J. rubens* secondary metabolites in hydrogel form for the first time. The results showed that the hydrogel was highly active against *C. tropicalis*. In a time-dependent death study, it was found that *J. rubens* PVA/PVP hydrogel stopped the development of *C. tropicalis* in the first 5 min. At the same time, *J. rubens* hydrogel has physicochemical properties that can be used actively in many areas, such as pathogen absorbent, drug coating, wound treatment, and functional food. *J. rubens*-based PVA/PVP hydrogel can be used in wound healing, development of cosmetic products, design of antimicrobial materials, and development of biomedical sanitary pads and other medical products. In the next study, it is planned to conduct in vitro biocompatibility analysis of the bioactive hydrogel, in vitro healing assay in *C. tropicalis*-infected wound (scratch migration assay), and in vivo healing assay in *C. tropicalis*-infected wound. Future in vivo studies are planned to investigate multi-infection caused by symbiotic relationships with other *Candida* members other than* C. tropicalis*.

## Data Availability

All experimental data and analyses of the study are included in this published article.
